# Supplementary material for: Predicting signatures of anisotropic resonance energy transfer in dye-functionalized nanoparticles
Source: RSC Adv. 2016 Oct 25;6(106):104648–56. doi: 10.1039/c6ra22433d (PMC5171219; doi:10.1039/c6ra22433d)
Supplement: Supplementary file 1 [file RA-006-C6RA22433D-s001.pdf]

Electronic Supplementary Material (ESI) for RSC Advances.
This journal is © The Royal Society of Chemistry 2016

Supporting information for: Predicting signatures of anisotropic resonance energy transfer in dye-functionalized nanoparticles

Gabriel Gil,[*,†,‡,§] Stefano Corni,[*,†] Alain Delgado,[¶,‖] Andrea Bertoni,[†] and Guido Goldoni[*,†,‡]

†*S3, CNR-Istituto Nanoscienze, Via Campi 213/A, 41125 Modena, Italy.*

‡*Dipartimento di Scienze Fisiche, Informatiche e Matematiche, Università degli Studi di Modena e Reggio Emilia, Modena, Italy.*

¶*Department of Physics, University of Ottawa, Ottawa, Canada.*

§*Instituto de Cibernética, Matemática y Física (ICIMAF), Havana, Cuba.*

‖*Centro de Aplicaciones Tecnológicas y Desarrollo Nuclear, Havana, Cuba.*

E-mail: gabrieljose.gilperez@nano.cnr.it; stefano.corni@nano.cnr.it; guido.goldoni@unimore.it

Contents: Molecular structure and TDM of Cy3B. Table of dielectric constants. Dielectric mismatch at the surface of the NP: excitonic states and TEF of the NP. Additional data in RET rates calculation. Experimental absorption and PL spectra of the dye and the NP. Master equations and time-dependent populations. Steady-state rates and spectra. Blocking and back-transfer effects. Octahedral tessellation. Average RET rates. Details on Figure 5 inset of the main text. Poisson distribution of the number of dyes per NP.

N.B.: We refer to the main text as m.t.

Molecular structure and TDM of Cy3B

The optimized geometry of Cy3B dye calculated using B3LYP/6-31G in vacuum, as described in the Methods section, is shown in Tables S1 and S2. The TDMs of the excited state corresponding to the first absorption peak of the dye, calculated through CAM-B3LYP/6-31G(d,p), are: (3.94, 7.95, -3.20) D in vacuum, (-5.07, 9.99, -3.14) D in water, and (-5.09, 10.02, -3.15) D in water with a nearby NP (vector moduli of 9.43 D, 11.64 D and 11.67 D, respectively). The latter vectors are consistent with the molecular structure presented in Tables S1 and S2. For the TDDFT-PCM excited states calculation of the dye in the presence of the NP, the nuclear center of charges of the dye was placed at a distance of 4.2 nm from the center of the NP, while all atomic positions are determined once the TDM, corresponding to the dye in water, is oriented radially with respect to the NP.

Table S1: Atomic cartesian coordinates (in angstroms) of Cy3B optimized geometry in vacuum (see Methods). To be continued in Table S2.

C	2.5339187152	0.8459649542	-0.5060287412
C	3.7651521728	0.8770531111	0.4418868715
C	4.9905386653	1.5136802092	-0.2697464141
C	4.0847396595	-0.5371426550	0.9338150811
C	4.3679595633	-1.6966084046	0.2255769637
C	4.5917138257	-2.9014948766	0.9241055158
C	4.8833373112	-4.1699349443	0.1247362299
C	4.4789269865	-5.4199381847	0.8640394090
O	3.3678035484	-5.8397675185	1.1313608573
O	5.6762786812	-6.0864252621	1.2938612137
N	5.4407065137	-7.2151130600	2.1035879404
C	5.3210763903	-7.0920942604	3.4929355577
O	5.3191757821	-6.0260398472	4.1136788986
C	5.1937529025	-8.5133661170	4.0269126553
C	5.1700897251	-9.4346128511	2.7795761136
C	5.3319011038	-8.5147715100	1.5729588889
O	5.3620005272	-8.7952871881	0.3778673829
C	4.5439068867	-2.9116315280	2.3285599258
C	4.2317011441	-1.7506656990	3.0519761217
C	3.9972704537	-0.5808264789	2.3356245216
N	3.6177619776	0.6959215056	2.8120474011
C	3.0823388850	0.9357260270	4.1613299445
C	3.2221791536	2.4138588500	4.5279256275
C	2.7288996033	3.3492146226	3.4290237534
O	1.2652412355	3.5561073988	3.5444466455
C	0.8617737293	4.6894629666	2.7299090269
C	-0.6528332042	4.6173012541	2.5055442320
C	-1.1121360531	5.8927540584	1.7910652738
N	-0.1866657316	6.2604297054	0.6938542095
C	1.0180186758	5.6933852969	0.4580369425
C	1.5566460095	4.7342717803	1.3587311668
C	2.5221715861	3.7958112432	1.0294081934
C	2.9981795093	2.8823602703	2.0154474349
C	3.4539765155	1.5959076904	1.7775162089
C	1.6623777388	6.3720041134	-0.7683704854
C	2.9870766091	7.0742039944	-0.3472384619
C	1.9198858915	5.4106982548	-1.9644473611
C	0.5861374113	7.3926441197	-1.1383482346
C	0.5310321972	8.3138683855	-2.1731637809
C	-0.6164416484	9.1084770431	-2.2744127366

Table S2: Continuing from Table S1.

C	-1.6871138999	9.0038503564	-1.3858705604
C	-1.6453669684	8.0679826434	-0.3424565400
C	-0.4956661520	7.2820744465	-0.2487045801
S	-0.7090582604	10.3578115725	-3.6909047724
O	-0.5048677967	9.3711624669	-5.0012596104
O	-2.2114749095	11.0129651311	-3.4836797522
O	0.5732344924	11.3497774131	-3.3781499056
H	1.6496510510	0.4580650292	0.0073635577
H	2.7493386620	0.1918614679	-1.3590477346
H	2.2988888486	1.8400256923	-0.8955215234
H	5.8791783447	1.4784785933	0.3699981297
H	4.7963156679	2.5570279232	-0.5365663336
H	5.2073565310	0.9622901462	-1.1891046474
H	4.3978847552	-1.6910783598	-0.8611072627
H	5.9450756651	-4.2416483821	-0.1349610890
H	4.3070107177	-4.1475037848	-0.8078233626
H	6.0410317966	-8.7165257566	4.6920085688
H	4.2845165277	-8.5896702942	4.6331112424
H	5.9786640956	10.1710439350	2.7784391120
H	4.2292941346	-9.9884699188	2.6805746046
H	4.7374257598	-3.8278458682	2.8797602692
H	4.1713974870	-1.7874348433	4.1333850384
H	2.0277796937	0.6266837078	4.1838122540
H	3.6356079037	0.3144513289	4.8717649843
H	2.6717829281	2.6261207862	5.4493718658
H	4.2803202453	2.6369673728	4.7127915421
H	3.2002754453	4.3327135331	3.5768365202
H	1.1032372251	5.6142102797	3.2862523354
H	-1.1767505585	4.5201352845	3.4614553372
H	-0.8768831149	3.7265632815	1.9084561708
H	-1.1431357377	6.7353031950	2.4878656551
H	-2.1113464236	5.7757765073	1.3639942717
H	2.8519832573	3.7156102353	0.0017723533
H	2.8232294933	7.7880417217	0.4654733578
H	3.3910058371	7.6225449998	-1.2044540781
H	3.7304982457	6.3353795931	-0.0207590179
H	1.0379263071	4.8023015985	-2.1881109178
H	2.7820969966	4.7535202912	-1.7975434845
H	2.1375471389	6.0156351088	-2.8503219821
H	1.3282626122	8.4364128501	-2.8992215552
H	-2.5304264109	9.6687229101	-1.5315754325
H	-2.4703282830	7.9774972062	0.3559769783

Tables of dielectric constants

Table S3: Static and dynamic dielectric constant of CdSe, ZnS, the homogeneous NP (volume-weighted averages) and water.

	CdSe	ZnS	NP	water
ϵ_s	9.29[S1]	8.31[S1]	8.85	78.39
ϵ_d	6.20[S1]	6.18[S2]	6.19	1.776

Dielectric mismatch at the surface of the NP: excitonic states and TEF

The Configuration Interaction approach for single excitonic states presented in Ref. S3 is valid for the case of no dielectric mismatch at the surface of the NP. In our case the NP is embedded in a different dielectric material (S3). Therefore, the exciton Hamiltonian (Eq. (7) ibid.), is substituted by $\hat{H} = \hat{H}^{(e)} + \Sigma(\mathbf{r}_e) + \hat{H}^{(h)} + \Sigma(\mathbf{r}_h) - V(\mathbf{r}_e, \mathbf{r}_h)$. $\hat{H}^{(e)}$ ($\hat{H}^{(h)}$) and $\mathbf{r}_e$ ($\mathbf{r}_h$) are the electron (hole) Hamiltonian and coordinate, respectively, (defined in Eq. (3) ibid.). $V(\mathbf{r}, \mathbf{r}')$ is the Green function of the generalized Poisson problem, i.e., $\nabla_{\mathbf{r}} \left\{\epsilon(\mathbf{r}) \cdot \nabla_{\mathbf{r}} V(\mathbf{r}, \mathbf{r}')\right\} = \delta(\mathbf{r} - \mathbf{r}')$, where $\epsilon(\mathbf{r})$ is the position-dependent dielectric constant. $\Sigma(\mathbf{r})$ is the self-energy associated with the latter problem.[S4] In our specific case, $\epsilon(\mathbf{r})$ is spherically symmetric and is defined piecewise as: $\epsilon(\mathbf{r}) = \epsilon_1$ for $r \leq R_{\mathrm{NP}}$, and $\epsilon(\mathbf{r}) = \epsilon_2$ for $r > R_{\mathrm{NP}}$, where ϵ_1 (ϵ_2) is the dielectric constant inside (outside) the NP, and R_{NP} the radius of the NP. The explicit expressions for $V(\mathbf{r}, \mathbf{r}')$ and $\Sigma(\mathbf{r})$ can be found in Ref. S5. $\Sigma(\mathbf{r})$ is introduced as an extra potential in the numerical solution of the electron and hole single-particle states.[S3]

The derivation of TEF for the case of no dielectric mismatch can be also found in Ref. S3. In the general case, we must substitute Eq. (15) and (17) (ibid.) by

$$\mathbf{E}\left(\mathbf{r};\mathbf{R}\right) = \nabla'[V(\mathbf{r},\mathbf{r}')]|_{\mathbf{r}'=\mathbf{R}}\,, \tag{1}$$

$$\mathbf{E}\left(\mathbf{r};\mathbf{R}\right) = \frac{1}{\epsilon_2}\sum_{l,m}\frac{4\pi}{(\epsilon+1)l+1}\, r^l\; Y_{lm}(\Omega)\; \mathbf{G}_{lm}(\mathbf{R}), \tag{2}$$

where $\mathbf{G}_{lm}(\mathbf{r}') = \nabla'\{r'^{-l-1}Y^*_{lm}(\Omega')\}$. Note that the last expression is only valid when $r \leq R_{\mathrm{NP}}$ and $R > R_{\mathrm{NP}}$, which is the case of interest: TEF generated by the NP at the position of the point-like TDM of dye (Eq. (5), m.t.).

Additional data in RET rates calculation

Table S4: Relevant quantities (spectral overlap, electronic coupling, RET/intrinsic decay lifetimes ratio, and RET rate) for each of the possible RET process. The dye-NP distance selected was $R = 4.23$ nm. This table is an extended version of Table 2 in the m.t.

D-A	J_{DA} (eV^{-1})	$\lvert V_{\mathrm{DA}}\rvert^2$ (eV/ns)	$\tau_{\mathrm{DA}}/\tau_{\mathrm{D}}$	k_{DA} (ns^{-1})
dye-NP	1.774	15.952	0.002	177.812
NP-dye	0.088	15.952	0.071	8.846
dye-dye	1.063	0.187	0.286	1.249

Experimental absorption and PL spectra of the dye and NP

Figure S1 shows the experimental absorption and PL spectra of the dye[S6] and the NP[S7] used to compute the spectral overlap factors $J_{\mathrm{M}\to\mathrm{NP}}$ and $J_{\mathrm{NP}\to\mathrm{M}}$ reported in Table S4 (Eq. (6) in the m.t.).

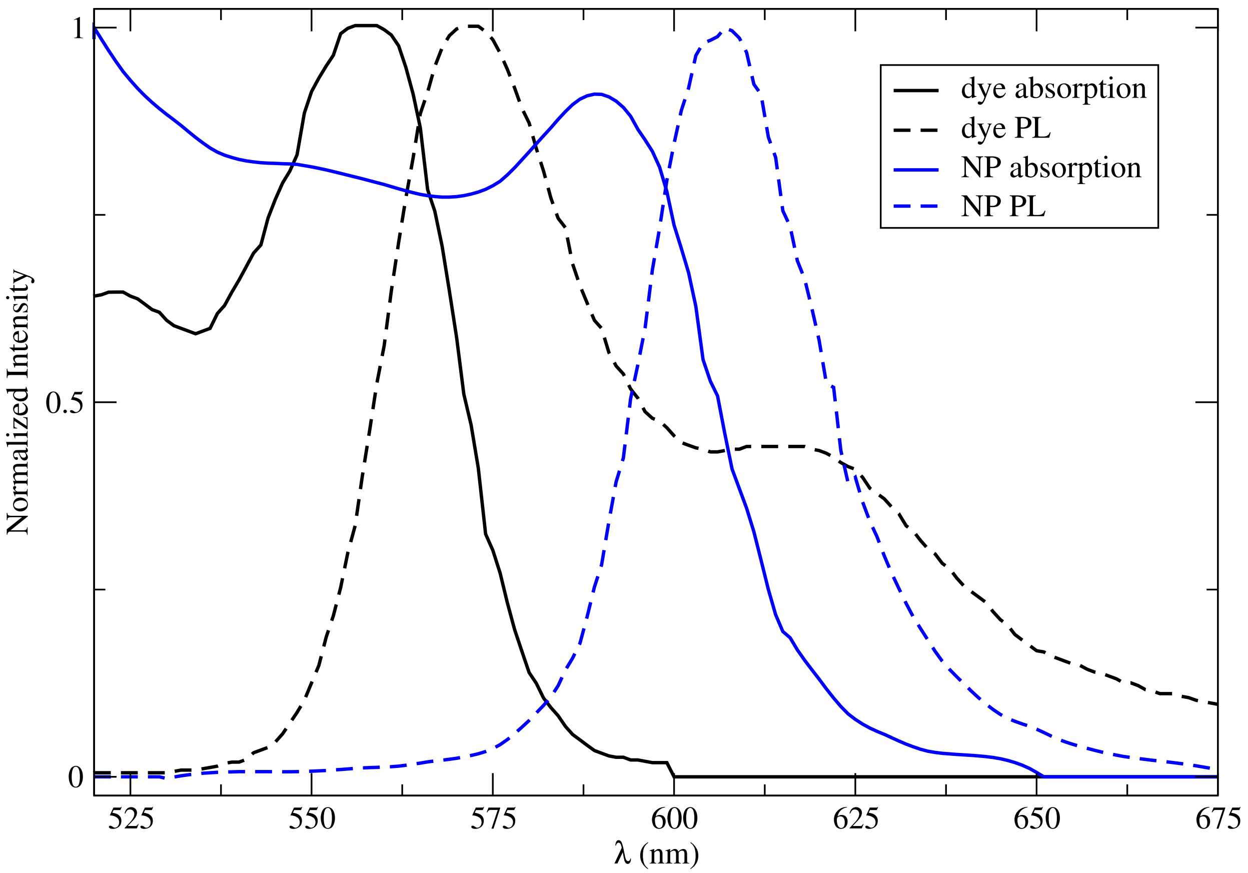

Figure S1: Experimental absorption and PL spectra of the dye (from Ref. S6) and NP (from Ref. S7).

Master equations and time-dependent populations

The kinetic model is based on the following set of master equations,

$$\begin{aligned}
\frac{dP_{\mathrm{M}_x}}{dt} =& 2k^{\mathrm{M}}_{abs}\sin^2\gamma\;(1-P_{\mathrm{M}_x}) + 4k_{\mathrm{MM}}(P_{\mathrm{M}_y}+P_{\mathrm{M}_z})(1-P_{\mathrm{M}_x}) + \\
& - 2k_{\mathrm{M}}P_{\mathrm{M}_x} - 4k_{\mathrm{MM}}P_{\mathrm{M}_x}(2-P_{\mathrm{M}_y}-P_{\mathrm{M}_z}) \quad \text{(3a)} \\
\frac{dP_{\mathrm{M}_y}}{dt} =& 4k_{\mathrm{MM}}(P_{\mathrm{M}_x}+P_{\mathrm{M}_z})(1-P_{\mathrm{M}_y}) + 2k_{\mathrm{NP}\to\mathrm{M}}P_{\mathrm{NP}_y}(1-P_{\mathrm{M}_y}) + \\
& - 2k_{\mathrm{M}}P_{\mathrm{M}_y} - 4k_{\mathrm{MM}}P_{\mathrm{M}_y}(2-P_{\mathrm{M}_x}-P_{\mathrm{M}_z}) - 2k_{\mathrm{M}\to\mathrm{NP}}P_{\mathrm{M}_y}(1-P_{\mathrm{NP}_y}-P_{\mathrm{NP}_z}) \quad \text{(3b)} \\
\frac{dP_{\mathrm{M}_z}}{dt} =& 2k^{\mathrm{M}}_{abs}\cos^2\gamma\;(1-P_{\mathrm{M}_z}) + 4k_{\mathrm{MM}}(P_{\mathrm{M}_x}+P_{\mathrm{M}_y})(1-P_{\mathrm{M}_z}) + 2k_{\mathrm{NP}\to\mathrm{M}}P_{\mathrm{NP}_z}(1-P_{\mathrm{M}_z}) + \\
& - 2k_{\mathrm{M}}P_{\mathrm{M}_z} - 4k_{\mathrm{MM}}P_{\mathrm{M}_z}(2-P_{\mathrm{M}_x}-P_{\mathrm{M}_y}) - 2k_{\mathrm{M}\to\mathrm{NP}}P_{\mathrm{M}_z}(1-P_{\mathrm{NP}_y}-P_{\mathrm{NP}_z}) \quad \text{(3c)} \\
\frac{dP_{\mathrm{NP}_y}}{dt} =& 2k_{\mathrm{M}\to\mathrm{NP}}P_{\mathrm{M}_y}(1-P_{\mathrm{NP}_y}-P_{\mathrm{NP}_z}) - k_{\mathrm{NP}}P_{\mathrm{NP}_y} - 2k_{\mathrm{NP}\to\mathrm{M}}P_{\mathrm{NP}_y}(1-P_{\mathrm{M}_y}) \quad \text{(3d)} \\
\frac{dP_{\mathrm{NP}_z}}{dt} =& k^{\mathrm{NP}}_{abs}\cos^2\gamma\;(1-P_{\mathrm{NP}_y}-P_{\mathrm{NP}_z}) + 2k_{\mathrm{M}\to\mathrm{NP}}P_{\mathrm{M}_z}(1-P_{\mathrm{NP}_y}-P_{\mathrm{NP}_z}) + \\
& - k_{\mathrm{NP}}P_{\mathrm{NP}_z} - 2k_{\mathrm{NP}\to\mathrm{M}}P_{\mathrm{NP}_z}(1-P_{\mathrm{M}_z}) \quad \text{(3e)}
\end{aligned}$$

where $\gamma = \arccos(\mathbf{e}\cdot\hat{z})$. NP-bright (NP-dark) configuration corresponds to $\gamma = 0$ ($\gamma = \pi/2$). The populations of the degenerate excited states of the NP with $\mathbf{d}^{(+)}_{\mathrm{NP}}$ or $\mathbf{d}^{(-)}_{\mathrm{NP}}$ are denoted as P_{NP_z} and P_{NP_y}, respectively. P_{M_x}, P_{M_y} and P_{M_z} correspond to the excited state populations of each of the dyes with $\mathbf{d}_{\mathrm{M}} \parallel \hat{x}$, $\hat{y}$, and $\hat{z}$, accordingly. The latter kinetic equations are specific for our prototypical NC with six dyes tethered to the NP in an octahedron configuration and coherent with the crystal symmetry of the NP lattice, as seen in m.t.

The time-dependent solutions of Eqs. (3) are shown in Fig. S2. We refer to the dyes located in the bright plane (dark axis) of the NP as bright-dyes (dark-dyes). Note that the steady-state populations are reached within the first 10 ns.

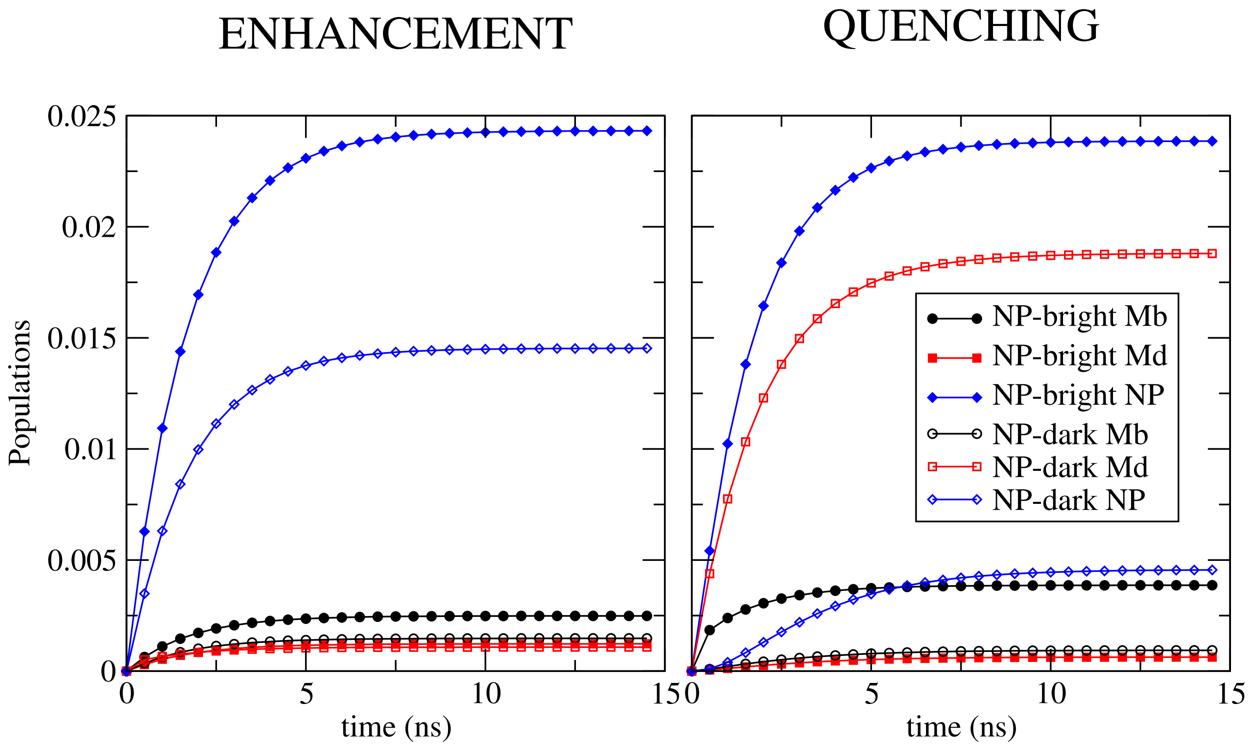

Figure S2: Time-dependent excited state populations of the bright-dyes, dark-dyes, and the NP in the enhancement/quenching ($R = 4.23$ nm / $R = 7.67$ nm) regimes for NP-bright/NP-dark configurations (Eqs. (3)). Mb and Md in legend stands for bright-dyes and dark-dyes, respectively.

Steady-state rates and spectra

The steady-state total emission rates of the dyes and the NP are defined by

$$c_{\mathrm{M}} = 2 \lim_{t\to\infty} k_{fl}^{\mathrm{M}} \left(P_{\mathrm{M}_x}(t) + P_{\mathrm{M}_y}(t) + P_{\mathrm{M}_z}(t) \right), \tag{4a}$$

$$c_{\mathrm{NP}} = \lim_{t\to\infty} k_{fl}^{\mathrm{NP}} \left(P_{\mathrm{NP}_y}(t) + P_{\mathrm{NP}_z}(t) \right), \tag{4b}$$

respectively, where the dependence on γ and R is understood. The total emission rates for NP-bright and NP-dark configurations, i.e., the coefficients entering in Eq. (7) of the m.t., are $c^b_{\mathrm{M}} \equiv c_{\mathrm{M}}(\gamma = 0)$ and $c^b_{\mathrm{M}} \equiv c_{\mathrm{M}}(\gamma = \pi/2)$.

In Fig. S3, we show the NP-bright/NP-dark spectra in the enhancement/quenching

regimes.

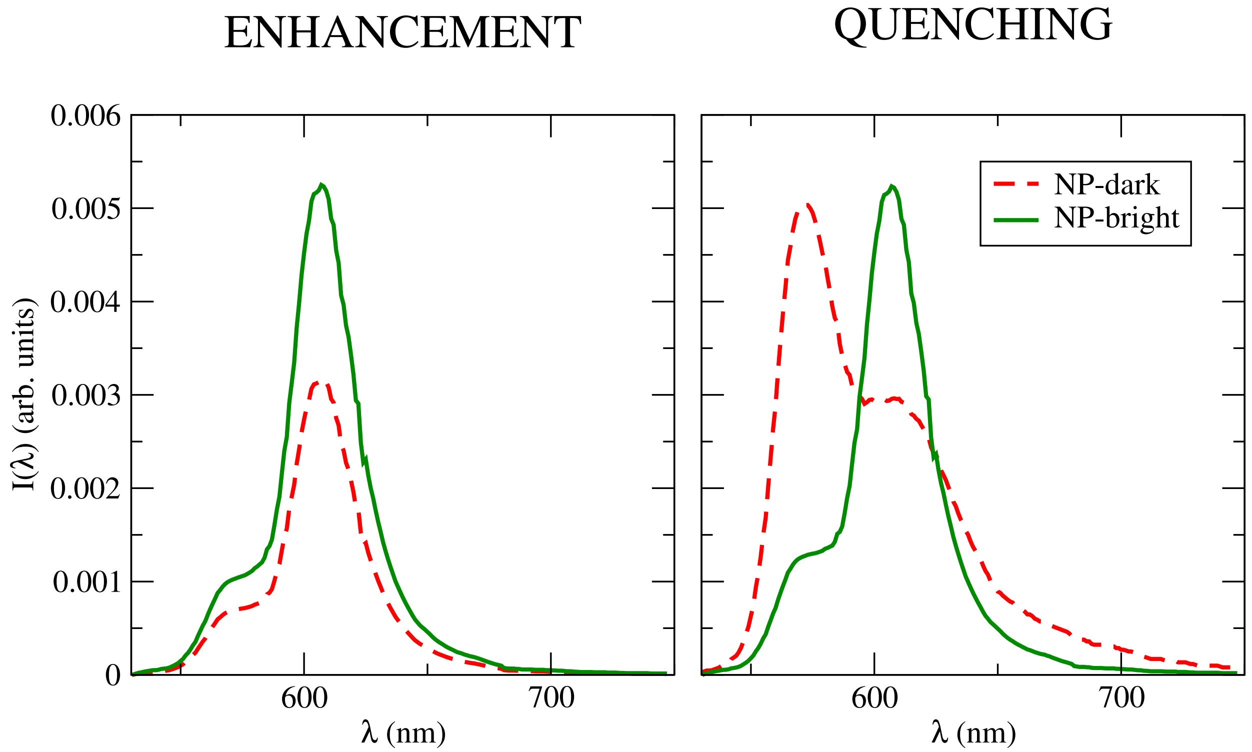

Figure S3: Calculated NP-bright and NP-dark spectra in the enhancement/quenching ($R = 4.23$ nm / $R = 7.67$ nm) regimes (cf. Figs. S2, and 3 in the m.t.).

We define as well the steady-state net RET rates $K_{\mathrm{M}\to\mathrm{NP}}$ and K_{MM} (capital letter K) as follows

$$\begin{aligned} K_{\mathrm{M}\to\mathrm{NP}} =& 2k_{\mathrm{M}\to\mathrm{NP}} P_{\mathrm{M}_z}(1 - P_{\mathrm{NP}_y} - P_{\mathrm{NP}_z}) + 2k_{\mathrm{M}\to\mathrm{NP}} P_{\mathrm{M}_y}(1 - P_{\mathrm{NP}_y} - P_{\mathrm{NP}_z}) \\ & - 2k_{\mathrm{NP}\to\mathrm{M}} P_{\mathrm{NP}_z}(1 - P_{\mathrm{M}_z}) - 2k_{\mathrm{NP}\to\mathrm{M}} P_{\mathrm{NP}_y}(1 - P_{\mathrm{M}_y}), \end{aligned} \tag{5a}$$

$$\begin{aligned} K_{\mathrm{MM}} =& 4k_{\mathrm{MM}} P_{\mathrm{M}_z}(1 - P_{\mathrm{M}_x}) + 4k_{\mathrm{MM}} P_{\mathrm{M}_y}(1 - P_{\mathrm{M}_x}) \\ & - 4k_{\mathrm{MM}} P_{\mathrm{M}_x}(1 - P_{\mathrm{M}_z}) - 4k_{\mathrm{MM}} P_{\mathrm{M}_x}(1 - P_{\mathrm{M}_y}) \end{aligned} \tag{5b}$$

where $P_{\mathrm{M}_{x,y,z}}$ and $P_{\mathrm{NP}_{y,z}}$ are steady-state populations. $K_{\mathrm{M}\to\mathrm{NP}}$ is the rate of RET from bright-dyes to NP, which is the only possible dye-NP RET, while K_{MM} is the rate of RET from bright-dyes to dark-dyes. Both $K_{\mathrm{M}\to\mathrm{NP}}$ and K_{MM} depend also on the polarization (e.g.,

NP-bright/NP-dark) through the populations (Fig. S2).

Equations (4) and (5) are specific for the prototypical NC with six dyes tethered to the NP in an octahedron configuration and coherent with the crystal symmetry of the NP lattice, as seen in m.t.

Table S5 show net RET and emission rates in the enhancement/quenching regimes for NP-dark/NP-bright configurations. It is remarkable that the dye excitations are preferentially transferred to the NP (i.e, $c_{\mathrm{M}} \ll K_{\mathrm{M}\to\mathrm{NP}}$) in all cases, except for the NP-dark configuration in the quenching regime, where intrinsic decay is a more efficient mechanism (i.e, $c_{\mathrm{M}} > K_{\mathrm{M}\to\mathrm{NP}}$). For NP-dark configuration in the enhancement regime, the transfer is accomplished in two steps: (i) the dye-dye RET from the photoexcited dyes to the dyes aligned with the NP bright plane, and (ii) the dye-NP RET from the latter intermediate dyes, as seen in Table S5. For NP-bright configuration, whether in the enhancement or the quenching regime, the dominant process is the direct dye→NP transfer from the photoexcited dyes. Finally, we note that, even filtering the NP PL, the enhancement ($c^b_{\mathrm{M}} > c^d_{\mathrm{M}}$) and the quenching ($c^b_{\mathrm{M}} > c^d_{\mathrm{M}}$) are still present, which indicate that those properties are specific of the dye PL (Eq. (7), m.t.).

Blocking and back-transfer effects

Tables S6 and S7 are equivalent to Table S5 in the condition of $k^{\mathrm{NP}}_{abs} = 0$ and $k_{\mathrm{NP}\to\mathrm{M}} = 0$, respectively. The latter are used as consistency checks of our interpretations. Indeed, we corroborate from Tables S6 and S7 that NP absorption and back-transfer are both necessary for obtaining the relative enhancement of dye PL in NP-bright with respect to NP-dark configuration (cf. Table S5). In other words, in the condition of no absorption (blocking effect suppressed) or no back-transfer, total emission rates indicate a quenching, i.e., $c^b_{\mathrm{M}} < c^d_{\mathrm{M}}$, at all distances.

We say that dye→NP RET process is blocked when the NP is already excited, and

therefore, cannot accept further excitations before decaying. This blocking is accounted for by factors $(1 - P_{\mathrm{NP}_y} - P_{\mathrm{NP}_z})$ in the NP absorption and dye→NP RET terms of the kinetic equations, i.e., Eqs. (3). Notice that it is more probable to have this blocking events if the NP is also excited by light absorption. As discussed in the m.t., the blocking effect reduces the probability of dye→NP RET, thus increasing the probability of dye de-excitation by intrinsic decay pathways. Back-transfer, i.e., NP→dye RET, also reduces the net flux of excitations from dyes to the NP, by contributing to the dyes excited state population, which ultimately leads to an increase of the probability of dye de-excitation by intrinsic decay pathways. In Tables S6 and S7 we show how the total emission rates of the dyes in the NP-bright configuration decrease when the NP absorption or back-transfer are switched off, as expected, ultimately eliminating the relative enhancement of the PL.

Table S5: Net dye→NP (red), and bright-dyes → dark-dyes (blue) RET rates, along with total dye emission rates for NP-dark and NP-bright configurations in the case of enhancement/quenching ($R = 4.23$ nm / $R = 7.67$ nm). The dye absorption rate is 3.0×10^{-2} ns^{-1}. Bright-dyes are the four dyes placed in the bright plane of the NP, while dark-dyes are the two dyes in the dark axis of the NP. In the schematics, NP is drawn as a large circle, whereas bright-dyes (dark-dyes) are represented by a single black dot on top (to the right) of the NP.

ENHANCEMENT		QUENCHING	
NP-dark	NP-bright	NP-dark	NP-bright
Net RET rates (ns^{-1})			
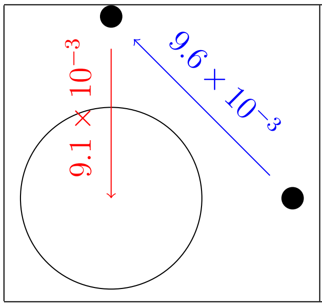	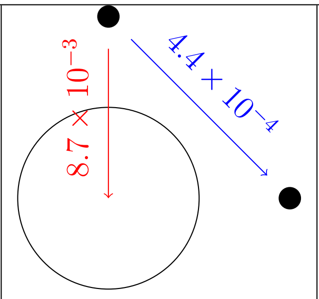	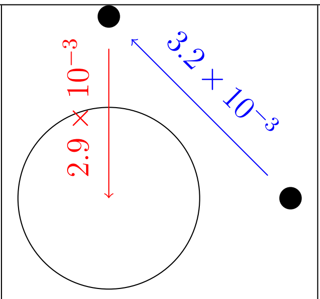	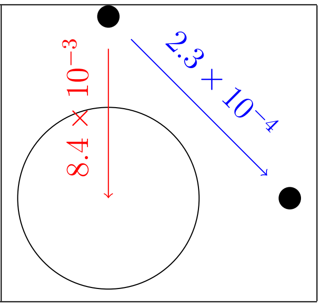
Total emission rates (ns^{-1})			
6.1×10^{-4}	8.9×10^{-4}	4.7×10^{-3}	1.1×10^{-3}

Table S6: Same as Table S5 for the case of $k_{abs}^{\mathrm{NP}} = 0$. No relative enhancement is found since blocking effect is suppressed.

~~ENHANCEMENT~~		QUENCHING	
NP-dark	NP-bright	NP-dark	NP-bright
Net RET rates (ns^{-1})			

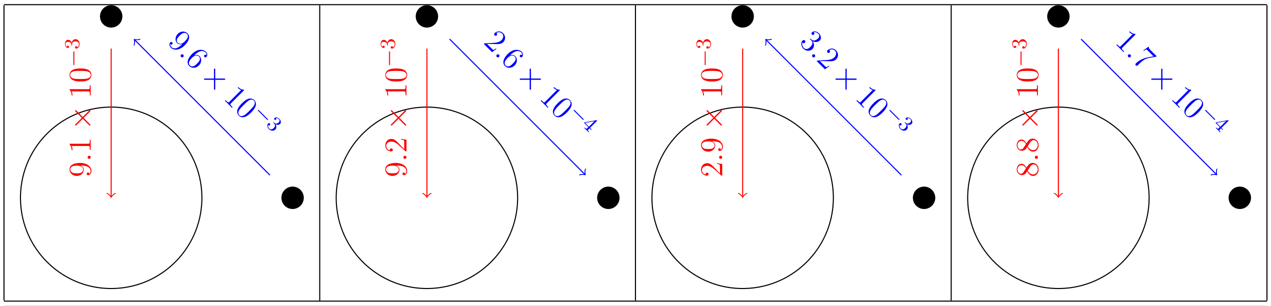

Total emission rates (ns^{-1})			
6.1×10^{-4}	5.3×10^{-4}	4.7×10^{-3}	8.1×10^{-4}

Table S7: Same as Tables S5 and S6 for the case of $k_{\mathrm{NP}\to\mathrm{M}} = 0$. No relative enhancement is found since back-transfer effect is suppressed.

~~ENHANCEMENT~~		QUENCHING	
NP-dark	NP-bright	NP-dark	NP-bright
Net RET rates (ns^{-1})			

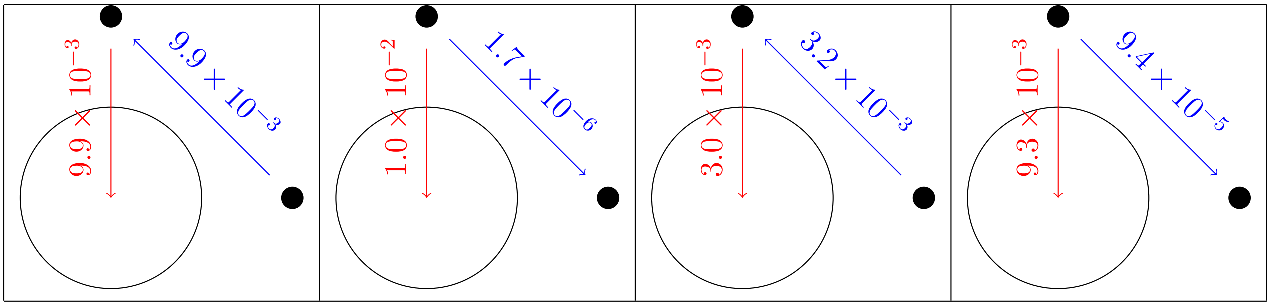

Total emission rates (ns^{-1})			
1.0×10^{-4}	3.5×10^{-6}	4.6×10^{-3}	4.5×10^{-4}

Octahedral tessellation.

To evaluate the dependence of the average dye-dye and dye-NP RET rates on N, we generate a collection of N-vertexes polyhedra circumscribed in a sphere of radius R representing the shell of dyes. Dyes are sitting at the vertexes of such polyhedra. In particular, we follow the rules for octahedral tessellation.[S8] The case of $N = 6$ is the one considered in the text, i.e., an octahedron. In general, the angular positions of the dyes are

$$\theta = \frac{\pi}{2}\frac{k}{s} \ , \tag{6a}$$

$$\phi = \begin{cases} 0 & \text{if } k = 0 \ , \\ \frac{\pi}{2}\frac{l}{k'} & \text{if } k \neq 0 \ ; \ \text{for } l = 0, \ldots, 4k' - 1 \ , \end{cases} \tag{6b}$$

for $k = 0, \ldots, 2s$, being $k' = \min(k, 2s - k)$, s an integer, and $N = 4s^2 + 2$. Notice that $N_{fn} = 6N/(N + 3)$ is the mean number of first neighbors in the N-vertexes polyhedron following octahedral tessellation. This sort of tessellation allows us to assure a close to uniform distribution of dyes in the surface of the NP for any N. Indeed, it is impossible to build a regular N-vertexes polyhedron for arbitrary N.[S9]

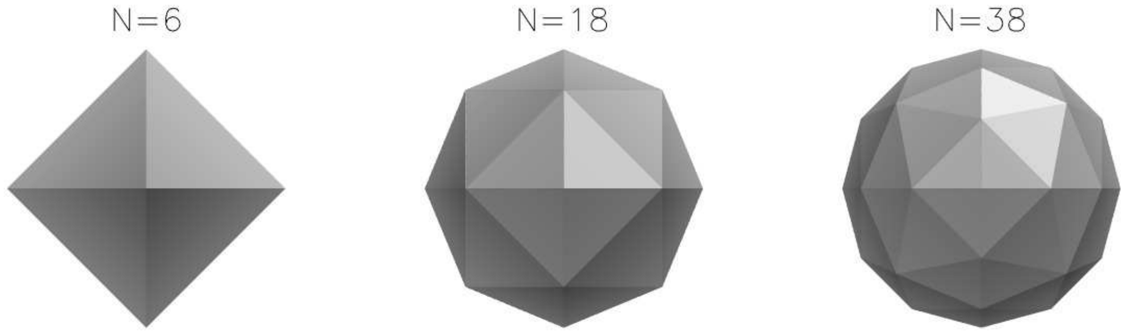

Figure S4: The octahedron ($N = 6$) and other two N-vertexes polyhedra generated by octahedral tessellation rules, corresponding to Eqs. (6).

From Eqs. (6), we obtain the dye-dye distances $R_{\rm MM}$ and the orientational factor $\kappa \equiv -\left(3(\mathbf{d}_{\rm M_1}\cdot\mathbf{R}_{\rm M_1M_2})(\mathbf{d}_{\rm M_2}\cdot\mathbf{R}_{\rm M_1M_2})/R^2_{\rm MM} - \mathbf{d}_{\rm M_1}\cdot\mathbf{d}_{\rm M_2}\right)/|\mathbf{d}_{\rm M}|^2$ in dye-dye RET rate (see Eq. (4) in the m.t.). Note that for $N = 6$, $\kappa = 3/2$.

Average RET rates

The average dye-dye and dye-NP RET rates are defined as

$$\Phi^{b/d}_{\rm MM} = \frac{\sum_i^N k^{\rm M}_{abs,b/d}(\theta_i,\phi_i)\sum_{j;J\neq i}^N k_{\rm MM}(\theta_i,\phi_i;\theta_j,\phi_j)}{\sum_i^N k^{\rm M}_{abs,b/d}(\theta_i,\phi_i)}, \tag{7a}$$

$$\Phi^{b/d}_{\rm M\to NP} = \frac{\sum_i^N k^{\rm M}_{abs,b/d}(\theta_i,\phi_i)\ k_{\rm M\to NP}(\theta_i,\phi_i)}{\sum_i^N k^{\rm M}_{abs,b/d}(\theta_i,\phi_i)}, \tag{7b}$$

respectively, where $k^{\rm M}_{abs,b/d}(\theta_i,\phi_i)$ is the absorption rate of the dye numbered i, while $N_{fn}(\theta_i,\phi_i)$ is the number of first neighbors around the site i. Integer i maps into a set of two integers, k and m, defining the polar and azimuthal angles given by Eqs. (6). b or d stands for NP-bright or NP-dark polarizations, respectively. $k^{\rm M}_{abs,b} = k^{\rm M}_{abs}\cos^2(\theta)$, whereas $k^{\rm M}_{abs,d} = k^{\rm M}_{abs}\sin^2(\theta)\ \cos^2(\phi)$. $k_{\rm MM}$ is dependent on a pair of angular positions corresponding to a pair of interacting dyes, and it is evaluated following Eqs. (2), (4) in the m.t., and Eqs. (6) here.

Details on Figure 5 inset of the main text

Figure 5 inset bars (m.t.) represent the region for which the PL quenching is possible, i.e., where $\Phi^b_{\rm M\to NP} > k_{\rm M}$, $\Phi^d_{\rm M\to NP} < k_{\rm M}$ and $\Phi_{\rm MM} < k_{\rm M}$. We only computed the maximum of $q(R)$ for the $N = 6$ case (Figure 3, m.t.). Nevertheless, we can estimate $q_{\max}$ by means of a simpler model, i.e., Eq. (9) (m.t.), and re-scale $q_{\max}$ in such a way that for $N = 6$ the result matches the actual value coming from Figure 3 (m.t.). The reported bar height in Figure 5

inset of m.t. for $N = 18$ is thus estimated. We take $\Phi^b_{\mathrm{M}\to\mathrm{NP}}$ in Eq. (9) (m.t.) at the middle point of the PL quenching region.

Poisson distribution of the number of dyes per NP

Let us, finally, consider that the dispersion of the number of dyes per NC follows a Poisson statistics, i.e., with probability distribution $P(n;N) = N^n e^{-N}/n!$, where now N is the expected value for the number of dyes per NC, and n is a particular realization of the number of dyes in a specific NC.[S10] We can then compute the expected quenching curve as

$$q(R;N) = \sum_{n=0}^{\infty} P(n;N)\ q(R;n), \tag{8}$$

where $q(R;n)$ is the quenching curve of a NC with n dyes. We get the approximated $q(R;n)$ from a linear interpolation of $q(R)$ for the cases with $n = 6$ and 18, i.e., $q(R;n) = (q(R;18) - q(R;6))/12 \times (n-6) + q(R;6)$. The latter $q(R;6)$ and $q(R;18)$ are quenching curves coming from a generalization of the model already presented in Eq. (9) of the m.t., i.e.,

$$q(n;R) = \frac{\Phi^b_{\mathrm{M}\to\mathrm{NP}}(n;R)}{(k_\mathrm{M} + \Phi^b_{\mathrm{M}\to\mathrm{NP}}(n;R))}\ \Theta(k_\mathrm{M} - \Phi_{\mathrm{MM}}(n;R))\ \Theta(k_\mathrm{M} - \Phi^d_{\mathrm{M}\to\mathrm{NP}}(n;R)), \tag{9}$$

where the explicit dependence on n and R comes from the rates. Heaviside theta functions in Eq. (9) restrict the result to the region in which our model is valid (see m.t. and previous paragraph). After the interpolation and the average on n, for $N = 6$ and $N = 18$ we obtain an almost identical curve to that of $n = 6$ and $n = 18$, i.e., $q(R;N) \approx q(R;n)$, as observed in Fig. S5.

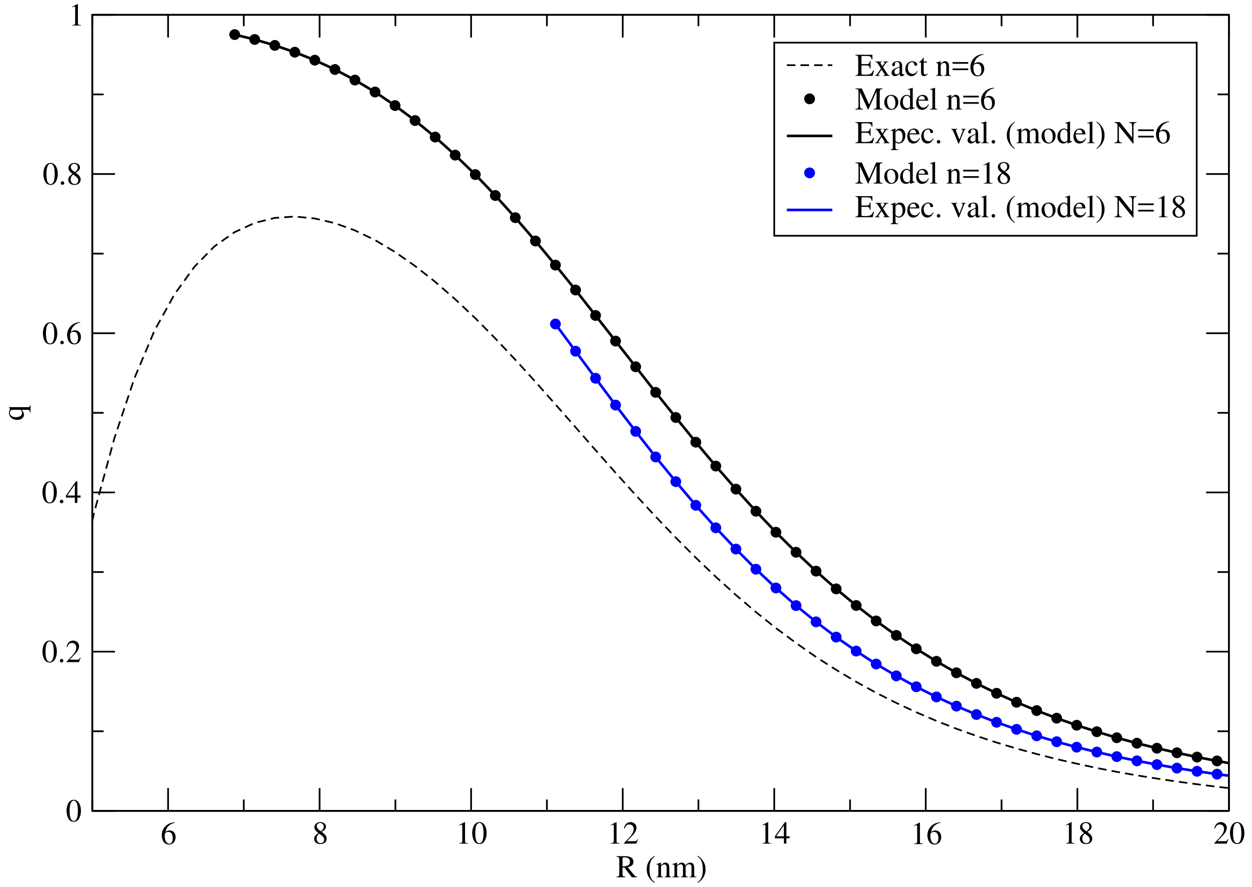

Figure S5: Quenching curves coming from the model in Eq. (9) for $n = 6$ and $n = 18$, along with the Poissonian expectation values for $N = 6$ and 18 (Eq. (8)). The quenching curve coming from the kinetic Eqs. (3) (i.e., $n = 6$), here called "exact", is also plotted as a reference.

References

(S1) Madelung, O. *Semiconductor Data Handbook*, 3rd ed.; Springer, 2004.

(S2) Khenata, R.; A., B.; Sahnoun, M.; Reshak, A. H.; Baltache, H.; Rabah, M. *Computational Materials Science* **2006**, *38*, 29–38.

(S3) Gil, G.; Corni, S.; Delgado, A.; Bertoni, A.; Goldoni, G. *The Journal of Chemical Physics* **2016**, *144*, 074101.

(S4) Banyai, L.; Koch, S. *Semiconductor Quantum Dots*; Atomic, Molecular and Optical Physics Vol. 2; World Scientific Publishing Co. Pte. Ltd., 1993.

(S5) Orlandi, A.; Goldoni, G.; Manghi, F.; Molinari, E. *Semiconductor Science and Technology* **2002**, *17*, 1302–1311.

(S6) Fluorophores.org. http://www.fluorophores.tugraz.at, 2015; [Online; accessed 04-December-2015].

(S7) Nabiev, I.; Rakovich, A.; Sukhanova, A.; Lukashev, E.; Zagidullin, V.; Pachenko, V.; Rakovich, Y.; Donegan, J.; Rubin, A.; Govorov, A. *Angewandte Chemie International Edition* **2010**, *49*, 7217–7221.

(S8) McReynolds, T., Blythe, D., Eds. *Advanced Graphics Programming Using OpenGL*; Elsevier Inc., 2005.

(S9) Duan, F., Guojun, J., Eds. *Introduction to Condensed Matter Physics*; World Scientific Publishing Co. Pte. Ltd., 2005.

(S10) Beane, G.; Boldt, K.; Kirkwood, N.; Mulvaney, P. *The Journal of Physical Chemistry C* **2014**, *118*, 18079–18086.
